# F18-FDG PET–CT in immune checkpoint inhibitor-associated acute interstitial nephritis: what is the diagnostic value?

**DOI:** 10.1093/ckj/sfag042

**Published:** 2026-02-12

**Authors:** Rafaella Maria da Cunha Lyrio, Victor D Cuenca Narvaez, Coraima Claudia Nava Chavez, Omar Al Refai, Michael Bold, Nelson Leung, Sandra M Herrmann

**Affiliations:** Department of Internal Medicine, Washington University, St. Louis, MO 63110, USA; Division of Nephrology and Hypertension, Mayo Clinic, Rochester, MN 55905, USA; Division of Nephrology and Hypertension, Mayo Clinic, Rochester, MN 55905, USA; Division of Nephrology and Hypertension, Mayo Clinic, Rochester, MN 55905, USA; Department of Nuclear Medicine, Mayo Clinic, Rochester, MN 55905, USA; Division of Nephrology and Hypertension, Mayo Clinic, Rochester, MN 55905, USA; Division of Nephrology and Hypertension, Mayo Clinic, Rochester, MN 55905, USA

**Keywords:** acute interstitial nephritis, acute kidney injury, FDG PET–CT, immune checkpoint inhibitors, immune-related adverse events

## Abstract

**Background:**

Immune checkpoint inhibitor-associated acute interstitial nephritis (ICI-AIN) requires a kidney biopsy for a definitive diagnosis. A recent study suggested that 2-deoxy-2-[18F] fluoro-D-glucose positron emission tomography-computed tomography (F^18^-FDG PET–CT) may offer a noninvasive alternative, but lack of kidney biopsies in all patients and a proper control group receiving ICI therapy remained important limitations. We conducted the first study addressing these limitations and examined whether PET–CT could differentiate biopsy-proven ICI-AIN from other causes of acute kidney injury (AKI) in patients on ICI therapy.

**Methods:**

This retrospective cohort study comprises 105 patients on ICI therapy who underwent F^18^-FDG PET–CT and had a kidney biopsy, along with data from a control group receiving ICIs without AKI. PET–CT scans were performed within 14 days before or 10 days after biopsy, and baseline PET-CTs obtained during ICI therapy before AKI, were reviewed by a blinded nuclear radiologist. Renal cortical standardized uptake values (SUV) were measured in five regions of interest, liver and blood-pool SUVs served as internal references.

**Results:**

Thirty-two patients were included (5 ICI-AIN, 7 AKI from other causes, 20 controls). Renal SUVs did not differ significantly among groups. In patients with serial PET-CTs, kidney SUVs tended to increase from baseline to AKI in ICI-AIN, but these changes were not statistically significant.

**Conclusions:**

In this biopsy-validated cohort, PET-CTs did not reliably distinguish ICI-AIN from other causes of AKI in patients receiving ICI therapy. Larger prospective studies are needed to validate these findings.

KEY LEARNING POINTS **What was known:**

- Definitive diagnosis of immune checkpoint inhibitors-associated acute interstitial nephritis (ICI-AIN) relies on kidney biopsy. However, biopsy is often not feasible.
- Prior studies suggested a potential diagnostic utility of PET–CT imaging, but the lack of biopsy confirmation and appropriate ICI-treated control groups limited the strength of their findings.

**This study adds:**

- In a biopsy-proven cohort of patients receiving ICI therapy, renal cortical standardized uptake values (SUVs) on F^18^-FDG PET–CT did not differ among patients with ICI-AIN, those with AKI from other causes, and ICI-treated controls without AKI.
- Furthermore, serial scans showed no significant increase in SUV during episodes of ICI-AIN.

**Potential impact:**

- F^18^-FDG PET–CT does not reliably distinguish ICI-AIN from other etiologies of AKI and should not be used in isolation for diagnostic decisions.
- Improved noninvasive tools for evaluating ICI-associated kidney injury are needed.

## Introduction

Immune checkpoint inhibitors (ICIs) have transformed cancer therapy by enhancing T-cell responses, but their expanding clinical use has raised concerns about immune-related adverse events (irAEs), particularly acute interstitial nephritis (AIN) [1]. Diagnosing AIN is challenging because kidney biopsy can be at times contraindicated or otherwise not feasible in patients with cancer, particularly due to coagulopathies or thrombocytopenia. Recent studies suggest that 2-deoxy-2-[18F]fluoro-D-glucose positron emission tomography-computed tomography (F^18^-FDG PET–CT), commonly used in cancer care, may offer a noninvasive alternative [2–6]. However, prior studies were small and lacked kidney biopsy confirmation in all patients with AKI and appropriate control groups of patients with cancer receiving ICI therapy. Here, we conducted the first and largest study with all patients having biopsy-proven ICI-AIN or AKI due to other causes addressing prior studies’ limitations and examined whether F^18^-FDG PET–CT can distinguish biopsy-proven ICI-associated AIN (ICI-AIN) from other causes of acute kidney injury (AKI) in patients receiving ICI therapy.

## Materials and methods

### Study design and participants

**Figure 1:**
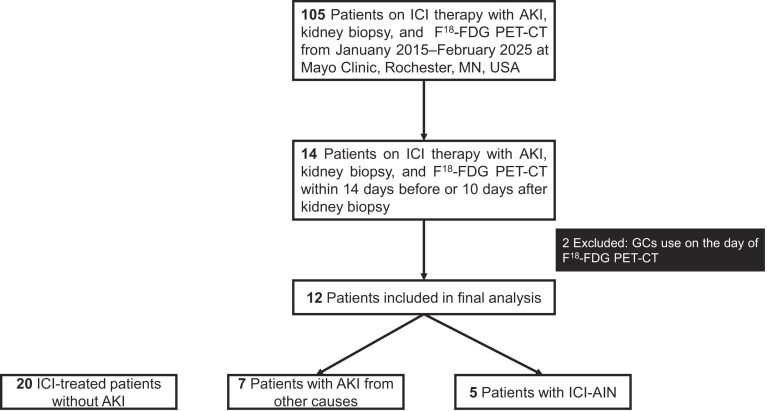
Flowchart of inclusion.

We conducted a retrospective cohort study of 105 patients receiving ICI therapy who developed AKI and underwent a kidney biopsy and F^18^-FDG PET–CT between 1 January 2015 and 1 February 2025 at Mayo Clinic, Rochester, MN, USA. Inclusion required the F^18^-FDG PET–CT performed within a predefined temporal window relative to the kidney biopsy, defined as within 14 days before or 10 days after the biopsy, to ensure temporal alignment between imaging and histopathologic findings. A control group consisting of ICI-treated patients without AKI at the time of F^18^-FDG PET–CT was also assembled; inclusion required a PET scan during ICI therapy (Fig. 1).

Patients were excluded from all groups if they had genitourinary cancer, lymphomatous kidney infiltration, or had received ≥7 days of glucocorticoids before the PET scan to minimize confounding of renal FDG uptake. Genitourinary malignancies and lymphomatous kidney infiltration can independently increase renal FDG uptake, while prolonged glucocorticoid exposure may attenuate inflammatory activity and alter PET–CT findings.

This study was approved by the Institutional Review Board of Mayo Clinic, with a waiver of informed consent.

### Data collection

Demographic characteristics, comorbidities including malignancy type, concurrent medication kidney function, and proteinuria were collected manually from electronic medical records before ICI therapy initiation. Both prior and concurrent extrarenal irAEs as documented by care providers were also collected.

Baseline creatinine was defined as the last stable serum creatinine (sCr) value before initiating ICI therapy. AKI was defined as an increase in serum creatinine (SCr) ≥50% from baseline, sustained for at least 48 hours. AKI severity was staged according to the KDIGO criteria. Renal recovery was defined as return of kidney function back to baseline or <25% from baseline at 3 months.

### F^18^-FDG PET–CT scan

F^18^-FDG PET–CT were performed within 14 days before or 10 days after kidney biopsy. In addition, within both AKI groups, we identified patients who had undergone a F^18^-FDG PET–CT scan before ICI therapy initiation.

A nuclear radiologist, blind to group assignment, reviewed the F^18^-FDG PET–CT scans. Five regions of interest (ROI), each 0.5 cm in diameter, were drawn in the renal cortex of each kidney, avoiding the collecting system, urine activity, and space-occupying lesions such as cysts. ROIs were selected to represent the upper, mid, and lower poles of each kidney. The mean standardized uptake value (SUV mean) was recorded for each ROI.

### Statistical methods

The data analysis was performed using IBM SPSS Statistics 28.0.0.0 (IBM Corp., Armonk, NY, USA) and respective graphs were obtained using GraphPad Prism version 10.4.2 (GraphPad Software, La Jolla, 195 CA, USA). Normality was assessed using the Shapiro–Wilk test. For parametric quantitative variables, data were presented as mean (standard deviation) or as percentages, and comparisons were evaluated using Student’s *t*-test or one-way ANOVA, followed by Tukey’s *post hoc* analysis. For non-parametric variables, data were presented as median (interquartile ranges), and comparisons were evaluated using Wilcoxon rank-sum test and Kruskal–Wallis test, followed by Dunn’s test for *post hoc* analysis.

In patients who underwent PET–CT both before and during AKI, paired comparisons of serum creatinine and SUV mean were performed. All other comparisons were unpaired. All tests were two-sided, and a *P* value <.05 was considered statistically significant.

## RESULTS

Thirty-two patients were included (five with ICI-AIN, seven with AKI from other causes, and 20 ICI-treated patients without AKI) (Fig. 1). Baseline characteristics are presented in Table 1. Age, gender, and race distributions were comparable across groups. The prevalence of chronic kidney disease was higher among patients with AKI from other causes compared to controls (*P* = .04). Similarly, serum kidney function parameters, including creatinine and estimated glomerular filtration rate (eGFR) were worse in this group.

**Table 1:** Baseline characteristics.

Variable	Control (*n* = 20)	AKI from other causes (*n* = 7)	ICI-AIN (*n* = 5)	*P* value
Age at treatment initiation, yrs, mean (SD)	66.7 (14.3)	65.4 (14.4)	71.4 (18.7)	.43
Female, *n* (%)	10 (50.0)	4 (57.1)	2 (40.0)	.84
Race				.16
White, *n* (%)	20 (100.0)	6 (85.7)	5 (100.0)	
Asian, *n* (%)	0 (0.0)	1 (14.3)	0 (0.0)	
Body mass index, mean (SD)	26.9 (5.1)	31.4 (7.2)	24.4 (3.8)	.08
Comorbidities, *n* (%)				
Hypertension	10 (50.0)	4 (57.1)	3 (60.0)	.90
Diabetes mellitus	3 (15.0)	4 (57.1)	1 (20.0)	.08
CHF	2 (10.0)	1 (14.3)	1 (20.0)	.50
COPD	3 (15.0)	1 (14.3)	0 (0.0)	.65
CKD	1 (5.0)	3 (42.9)^a^	2 (40.0)	**.04**
CKD etiology—hypertension	1 (100.0)	0 (0.0)	0 (0.0)	.73
CKD etiology—diabetes mellitus	0 (0.0)	3 (100.0)^a^	0 (0.0)	**.003**
CKD etiology—other/unknown	0 (0.0)	0 (0.0)	2 (66.7)^b^	**.003**
Malignancy, *n* (%)				
Melanoma	2 (10.0)	1 (14.3)	1 (20.0)	.82
Lung adenocarcinoma	8 (40.0)	1 (14.3)	2 (40.0)	.45
Lung squamous cell	3 (15.0)	0 (0.0)	0 (0.0)	.37
Hodgkin lymphoma	0 (0.0)	1 (14.3)	0 (0.0)	.16
Non-Hodgkin lymphoma	1 (5.0)	0 (0.0)	0 (0.0)	.73
Other	6 (30.0)	4 (57.1)	2 (40.0)	.44
Baseline sCr, (mg/dl), mean (SD)	0.85 (0.22)	1.38 (0.30)^a^	1.17 (0.41)	**.005**
Baseline eGFR (ml/min/1.73 m²), mean (SD)	84.1 (19.4)	48.7 (11.6)^a^	66.0 (27.0)	**.001**
eGFR ≥90	8 (40.0)	0 (0.0)	2 (40.0)	.13
eGFR 60–89	10 (50.0)	1 (14.3)	0 (0.0)	.05
eGFR 45–59	2 (10.0)	3 (42.9)	1 (20.0)	.16
eGFR 30–44	0 (0.0)	3 (42.9)^a^	2 (40.0)^b^	**.007**
AKI Stage 1	N/A	4 (57.1)	3 (60.0)	.92
AKI Stage 2	N/A	1 (14.3)	0 (0.0)	.68
AKI Stage 3	N/A	2 (28.6)	2 (40.0)	.68

Data are shown as mean (SD) and *n* (%). Abbreviations: CHF, chronic heart failure; COPD, chronic obstructive pulmonary disease.

^a^AKI from other causes vs control. CKD: *P =* 0.042; CKD-DM: *P =* 0.01; Baseline SCr: p 0.005; eGFR: *P =* 0.001; eGFR 30–44: *P =* 0.01.

^b^ICI-AIN vs control. CKD-Other: *P =* 0.03; eGFR 30–44: *P =* 0.04.

Detailed characteristics and clinical features of the five ICI-AIN patients are shown in Tables 2 and 3. Interestingly, all patients had prior or concomitant extrarenal irAEs, and none exhibited pyuria. Kidney biopsies revealed diffuse interstitial inflammatory infiltrates in three patients, while the other two demonstrated chronic interstitial nephritis. Among the four patients who underwent PET–CT both at baseline and near the time of AIN diagnosis, three showed diffuse acute interstitial infiltrates, and one had focal inflammation with areas of arteritis. Tubulitis was identified in four cases, with severity ranging from mild to focal mild-to-severe. In one biopsy, tubulitis was noted but not formally graded.

**Table 2:** Detailed characteristics of ICI-AIN patients.

						Extrarenal irAEs	
Number	Age/sex	Malignancy	ICI	sCr (mg/dL) BL/peak/nadir	Proteinuria(UPOS)	prior to AKI	Concomitant with AKI
1	40/F	Melanoma	Pem	0.80/2.50/1.00	0.57	Thyroid disease	None
2	86/M	Head and neck cancer	Cem	1.67/2.45/1.61	N/A	Thyroid disease, Hepatitis	None
3	83/M	Lung adeno	Pem	1.53/2.24/1.82	0.29	None	Thyroid disease
4	69/M	Lung adeno	Pem	0.81/1.42/1.38	1.05	Thyroid disease	None
5	79/F	Mesothelioma	Pem	1.04/3.77/1.43	0.82	Rash,Arthalgias	None

Abbreviations: BL, baseline; UPOS, urine protein-to-creatinine ratio (spot); adeno, adenocarcinoma; Pem, pembrolizumab; Cem, cemiplimab; N/A, not available.

**Table 3:** Clinical features of ICI-AIN patients.

Number	Biopsy-proven AIN?	Combination ICI therapy?^a^	ConcurrentPPI use?	Prior orconcomitantextrarenal irAE?	Pyuria?^b^	Treated with GCs?	Kidney recovery with GCs?
1	Yes	No	Yes	Yes	No	Yes	Yes
2	Yes	No	No	Yes	No	Yes	No
3	Yes	No	No	Yes	No	Yes	Yes
4	Yes	No	No	Yes	No	No*	N/A
5	Yes	No	No	Yes	No	Yes	No

Abbreviations: PPI, proton pump inhibitor; GCs, glucocorticoids; NA, not available.

a Refers to simultaneous treatment with a CTLA-4 inhibitor and a PD-1 or PD-L1 inhibitor.

b Pyuria was defined as ≥10 WBCs per high-power field.

* Steroids were not considered after renal biopsy, as this patient’s renal function was stable over the last 3 months.

Clinical features of the seven patients with AKI from other causes are summarized in Table 4. Kidney biopsies demonstrated acute tubular injury (ATI) in five patients, whereas the other two exhibited arteriosclerosis.

**Table 4:** Clinical features of patients with AKI from other causes.

Number	Age/sex	NSAIDs	PPI	Antibiotic	Etiology of AKI—biopsy proven
1	38/M	No	No	No	Acute tubular necrosis/injury (ATN/ATI).
2	61/F	No	No	No	Acute tubular necrosis/injury (ATN/ATI).
3	66/F	No	No	No	Nodular diabetic glomerulosclerosis.Moderate arteriosclerosis and arteriolar hyalinosis. Mild tubular atrophy interstitial fibrosis with chronic inflammation. Global glomerulosclerosis (7 of 36 glomeruli, 19%).
4	71/M	No	No	No	Arteriosclerosis, severe, with atheromatous emboli. Diffuse diabetic glomerulosclerosis.
5	66/M	No	Yes	Yes	Acute tubular necrosis/injury (ATN/ATI).
6	70/F	No	No	No	Acute tubular necrosis/injury (ATN/ATI).
7	86/F	No	No	Yes	Acute tubular necrosis/injury (ATN/ATI).

Abbreviations: ATN, acute tubular necrosis; ATI, acute tubular injury; NSAIDs, non-steroidal anti-inflammatory drugs; PPI, proton pump inhibitor.

**Figure 2:**
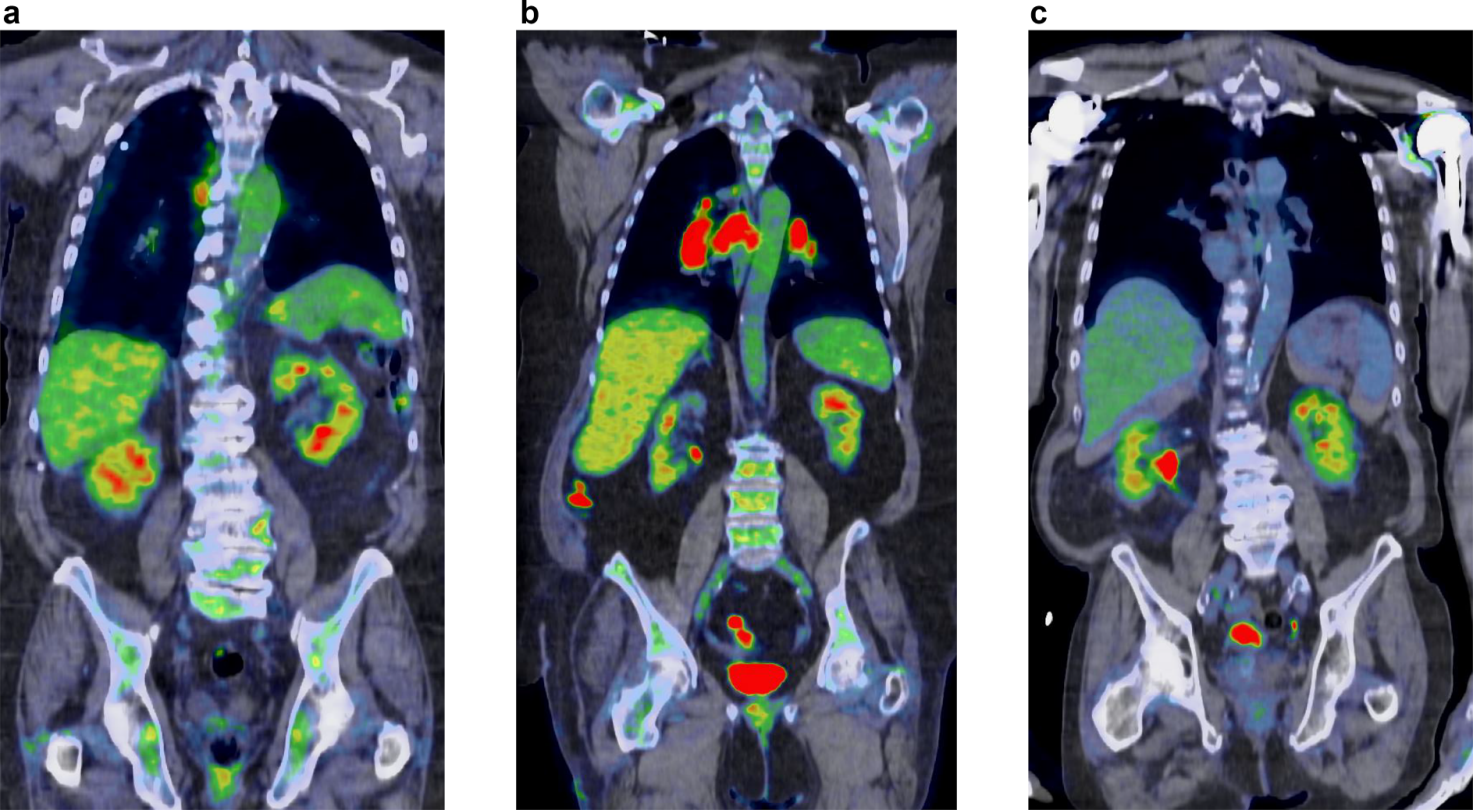
Representative F^18^-FDG PET–CT scans of study groups. (a) Control group. (b) AKI from other causes. (c) ICI-AIN.

Patients with ICI-AIN and AKI from other causes had significantly higher serum creatinine levels at the time of PET scan study compared with controls without AKI. Reference tissue SUVs (liver and blood pool) and renal cortical SUVs were similar among groups, with no significant differences (*P* = .29, .18, and .70, respectively) (see Table 5 and Fig. 2).

**Table 5:** SUV metrics.

Variable	Control (*n* = 20)	AKI from other causes (*n* = 7)	ICI-AIN (*n* = 5)	*P* value
sCr (mg/dl) at the time of PET scan, median (IQR)	0.91 (0.66, 1.09)	2.93 (2.05, 4.27)^a^	2.24 1.68, 3.07)^b^	<.001
Liver SUV, median (IQR)	2.55 (2.13, 2.80)	3.00 (2.60, 3.20)	2.60 (2.35, 3.05)	.29
Blood-pool SUV, median (IQR)	1.65 (1.40, 2.10)	2.10 (1.80, 2.30)	1.70 (1.60, 2.00)	.18
Kidney SUV, median (IQR)	3.16 (2.88, 3.52)	2.85 (2.59, 3.52)	3.01 (2.68, 4.13)	.70

Abbreviations: AIN, acute interstitial nephritis.

^a^ *P* < .05 for group AKI from other causes vs control in *post hoc* test adjusted for multiple comparisons.

^b^ *P* < .05 for group ICI-AIN vs control in *post hoc* test adjusted for multiple comparisons.

For patients with PET–CT scans available both before ICI therapy initiation and during the AKI diagnostic window, changes in serum creatinine and SUV values were assessed (Table 6). In both AKI groups, serum creatinine levels increased from baseline to the AKI episode. However, this difference only reached statistical significance in patients with AKI from other causes [median 1.50 (1.10–1.64) vs 3.23 (1.96–4.72) mg/dl, *P* = .03]. Conversely, kidney SUVs tended to decrease during an AKI episode in patients with AKI from other causes compared to baseline [median 3.10 (2.96–3.59) vs 2.84 (2.53–3.54), *P* = .06] whereas no significant change was observed in ICI-AIN patients [median 2.72 (2.57–3.12) vs 3.43 (2.66–4.28), *P* = .13]. Liver and blood-pool SUVs remained stable across time points in both groups.

**Table 6:** SUV metrics over time.

	AKI from other causes (*n* = 6)			ICI-AIN (*n* = 4)		
Variable	Baseline	AKI episode	*P* value	Baseline	AKI episode	*P* value
sCr (mg/dl), median (IQR)	1.50 (1.10, 1.64)	3.23 (1.96, 4.72)	.03	0.98 (0.83, 1.45)	2.32 (1.63, 3.41)	.13
Liver SUV, median (IQR)	2.95 (2.58, 3.20)	2.80 (2.45, 3.13)	.72	2.50 (2.35, 2.88)	2.65 (2.30, 3.23)	.63
Blood-Pool SUV, median (IQR)	2.10 (1.83, 2.50)	2.10 (1.70, 2.40)	.72	1.95 (1.75, 2.08)	1.80 (1.55, 2.05)	.75
Kidney SUV, median (IQR)	3.10 (2.96, 3.59)	2.84 (2.53, 3.54)	.06	2.72 (2.57, 3.12)	3.43 (2.66, 4.28)	.13

Abbreviations: AIN, acute interstitial nephritis; sCr, serum creatinine.

## DISCUSSION

This study rigorously assessed, for the first time, the diagnostic utility of F^18^-FDG PET–CT in differentiating patients with only biopsy-proven ICI-AIN from those with other causes of AKI, as well as from ICI-treated control patients without AKI. The results demonstrated that renal cortical SUVs were not significantly different between patients with ICI-AIN and those with AKI from other causes. Even among patients with serial PET–CT scans, changes in kidney SUVs from baseline to AKI episodes in ICI-AIN patients did not reach statistical significance. Within the ICI-AIN group, one patient had received a short course of corticosteroids, but discontinued therapy due to intolerance. Because AKI persisted and worsened, a kidney biopsy was performed. This patient had the lowest kidney SUV among the five ICI-AIN patients, which might have reduced the overall group mean. In patients who underwent serial PET-CTs obtained before and during AKI, longitudinal analyses showed a numerical trend toward decreasing kidney SUVs in those with AKI from other causes and increasing kidney SUV in those with ICI-AIN, but these changes did not reach statistical significance. However, it is important to note that these observations were based on a small number of patients, four ICI-AIN patients, limiting the ability to draw firm conclusions.

Although prior studies have reported higher SUVs in patients who developed ICI-AIN when compared with baseline PET-CTs obtained before kidney injury, we did not observe significant differences in our cohort [2–6]. Notably, PET-CTs in our study were interpreted by one of the same nuclear radiologists, and followed the same imaging protocol used in the Gupta *et al*. study, for which our institution provided the largest sample of ICI-AIN cases [3]. We believe this discrepancy may reflect limited statistical power and, more likely, the limited reliability of PET imaging in distinguishing ICI-AIN from other etiologies of AKI at a single time point, particularly in a cohort in which all patients were receiving ICI therapy and all AKI cases were biopsy-proven. Most of ICI-AIN biopsies in our cohort showed diffuse interstitial inflammation, with tubulitis present in nearly all cases. This pattern suggests that PET–CT may have limited sensitivity for detecting the full spectrum of ICI-AIN. In addition, to further decrease potential confounders, we also measured liver and blood-pool SUVs as internal references to control systemic variability. Using these internal references, we ensure that any observed differences in kidney SUVs are attributable to renal pathology itself, rather than systemic factors. Moreover, by including only biopsy-proven cases, we minimize misclassification bias, and this is the first study in which all patients, including controls, were on ICI therapy, which is important as the inflammatory response could be different in this ICI-treated population vs non-ICI-treated.

Our study has several limitations. First, the relatively small number of patients, whose PET–CT scans were obtained as part of routine cancer care rather than through a standardized research protocol, may have introduced variability in imaging timing and technical parameters and reduced statistical power. Second, as a single–center study, the generalizability of our findings may be limited. Finally, because this was a hypothesis–generating study and was not powered to detect subtle differences in SUV values, the negative findings are best interpreted as reflecting the limited discriminatory capacity of PET–CT rather than the absence of renal inflammation. However, the study has important strengths not addressed before. All AKI cases were biopsy proven, minimizing misclassification and strengthening the validity of the findings. The inclusion of ICI-treated controls without AKI addresses a major limitation of prior studies. In addition, PET–CT scans were interpreted by a blinded nuclear radiologist, reducing the potential for bias.

In summary, while F^18^-FDG PET–CT remains valuable for oncologic indications, our findings indicate that its utility in diagnosing ICI–AIN is limited. These results underscore the ongoing need for more reliable noninvasive diagnostic tools and reinforce the critical role of kidney biopsy in this challenging clinical scenario. Larger, prospective studies using standardized imaging protocols are needed to further evaluate the role of PET–CT and other noninvasive modalities in diagnosing ICI-AIN. Future research should explore imaging techniques that may offer greater sensitivity and specificity for immune-related kidney injury.

## Data Availability

The data underlying this article will be shared on reasonable request to the corresponding author.
